# Striking reduction in neurons and glial cells in anterior thalamic nuclei of older patients with Down syndrome

**DOI:** 10.1016/j.neurobiolaging.2018.11.009

**Published:** 2019-03

**Authors:** James C. Perry, Bente Pakkenberg, Seralynne D. Vann

**Affiliations:** a School of Psychology, Cardiff University, Cardiff, UK; b Research Laboratory for Stereology and Neuroscience, Copenhagen University Hospital, Denmark and Institute of Clinical Medicine, Faculty of Health, University of Copenhagen, Bispebjerg, Copenhagen, Denmark

**Keywords:** Down syndrome, Diencephalon, Anterior thalamus, Stereology, Dementia, Alzheimer's disease

## Abstract

The anterior thalamic nuclei are important for spatial and episodic memory, however, surprisingly little is known about the status of these nuclei in neurological conditions that present with memory impairments, such as Down syndrome. We quantified neurons and glial cells in the anterior thalamic nuclei of four older patients with Down syndrome. There was a striking reduction in the volume of the anterior thalamic nuclei and this appeared to reflect the loss of approximately 70% of neurons. The number of glial cells was also reduced but to a lesser degree than neurons. The anterior thalamic nuclei appear to be particularly sensitive to effects of aging in Down syndrome and the pathology in this region likely contributes to the memory impairments observed. These findings reaffirm the importance of examining the status of the anterior thalamic nuclei in conditions where memory impairments have been principally assigned to pathology in the medial temporal lobe.

## Highlights

• Volume of anterior thalamus is markedly reduced in older patients with Down syndrome.
• Number of neurons in anterior thalamus are substantially reduced.
• Number of glial cells in anterior thalamus are substantially reduced.

## 1 Introduction

Down syndrome (DS; trisomy 21) is the most common chromosomal disorder, affecting 1 in 1000 live births in the UK (Wu and Morris, 2013). A common feature of DS is learning disabilities with accompanying language and memory impairments. Over recent years, there has been a noticeable increase in life expectancy for adults with DS, with the median life expectancy in England and Wales currently at 58 years (Wu and Morris, 2013). This increase in lifespan has resulted in a concomitant increase in the number of adults with DS affected by dementia, particularly Alzheimer's disease (AD), which is considerably more prevalent in adults with DS than the general population (e.g., Zigman and Lott, 2007). Given the increased longevity of patients with DS, it has become increasingly important to understand the underlying changes that occur with age and dementia in this condition.

Research into mnemonic impairments in DS has typically focused on medial temporal lobe structures, which are smaller in patients with DS even when overall brain size is taken into account (Carducci et al., 2013, Kemper, 1991, Krasuski et al., 2002, Pinter et al., 2001). However, there is an increasing realization that it is necessary to look beyond the hippocampal formation in conditions that present with memory impairments. In doing so, the anterior thalamic nuclei (ATN) become an obvious target because of their connections with the hippocampal formation and their importance for memory (Dillingham et al., 2015, Harding et al., 2000, Jankowski et al., 2013, Van der Werf et al., 2003, Wolff and Vann, 2019). The presence of neurofibrillary tangles and plaques in the ATN of patients with AD further highlights the potential relevance of this structure to DS (Aggleton et al., 2016, Braak and Braak, 1991a, Rub et al., 2016). Despite these potential links, there is very little currently known about the status of the ATN in patients with DS. Neuroimaging studies have only focused on the thalamus as a whole (e.g., Annus et al., 2017, Teipel et al., 2004), which does not take into account the structural and functional heterogeneity in the thalamus. Owing to their size and location, it is difficult to obtain accurate measures of ATN volume using current neuroimaging techniques, which makes postmortem assessments all the more important. Moreover, postmortem tissue enables examination at the cellular level in addition to volumetric measures. Therefore, stereological counts of neurons and glial cells were made in the ATN of four aged female brains with DS and six age-matched controls.

## 2 Method

### 2.1 Patient characteristics

Brains from four female patients with DS (mean age 69 years; range 61–80 years) were provided by the Netherlands Brain Bank and six female age-matched controls (mean age 71 years; range 60–85 years) were obtained after death according to Danish law regarding the use of autopsied human tissue in research. The diagnosis of DS was based on the characteristic physical appearance of patients and presence of intellectual disability as postmortem chromosomal analysis was not available (see Table 1 for clinical data).

**Table 1:** Participant clinical details

Group	Sex	ID#	Age (y)	Height (cm)	Hemisphere (g)	Country	Cause of death	PMI (h)	Braak score
Controls	F	C1	75	159	575	DK	AMI	24:00	
	F	C2	85	159	590	DK	AMI	24:00	
	F	C3	64	154	620	DK	AMI	24:00	
	F	C4	60	161	520	DK	AMI	48:00	
	F	C5	70	169	667	DK	AMI	24:00	
	F	C6	74	160	590	DK	AMI	24:00	
Mean			71.3	160.3	594				
DS	F	P1	61	-	444	NED	Pneumonia	05:30	VI
	F	P2	64	148	371	NED	Pneumonia	04:00	V
	F	P3	70	162	387	NED	Ileus	09:00	VI
	F	P4	80	-	537	NED	AMI/pneumonia	06:00	V
Mean			68.8	155	435				

Key: AMI, acute myocardial infarction; DK, Denmark; DS, Down syndrome; NED, the Netherlands; PMI, postmortem interval.

Patient 1 (P1) was first noted as having seizures at the age of 36 years with an EEG showing mild diffuse cerebral disorder with focal abnormalities in both temporal regions; the patient was treated with antiepileptic medication. When she was 53 years old, patient 1 had been free from seizures for many years but started showing the first symptoms of dementia. Two years after the onset of dementia symptoms, she was admitted to a nursing home where she gradually deteriorated both mentally and physically. A year before death, she suffered from several epileptic seizures. The postmortem report detailed numerous senile plaques and neurofibrillary tangles within the frontal, temporal, parietal, and occipital lobes and the hippocampus. The substantia nigra showed very little, if any, cell loss and the pons, medulla oblongata, and cerebellum all appeared normal.

Patient 2 (P2) functioned at a moderate level for most of her life, working in a social working facility for almost 30 years until the age of 50 years. At the age of 57 years, she was admitted to a home because of increasing problems in taking care of herself, often getting lost, suffering from mood swings, and having difficulty taking care of personal needs. At the age of 60 years, it was thought she had between stage 3 and stage 4 AD and she started to have minor epileptic insults. EEG showed a diffuse abnormality. According to the postmortem neuropathology report, the brain was severely atrophied with numerous plaques and tangles across all lobes and in the hippocampus. The pons, putamen, medulla oblongata, and cerebellum showed no abnormality.

Patient 3 (P3) was diagnosed with epilepsy at the age of 26 years. An EEG carried out when the patient was 37 years showed diffuse irregular activity. She was admitted into a home when she was 44. At the age of 60, the patient had a marked decrease in general functioning with the likely diagnosis of AD, and at 64 years, she was thought to have stage 2 AD. A report from the following year stated that she was unable to differentiate between members of her family and nursing home staff and she was disoriented in time and place with difficulties in communication. In the last 2 months before death, a cognitive assessment showed her linguistic and communicative skills to be severely disturbed as were her memory function, orientation, and concentration. The postmortem neuropathological report identified numerous tangles and plaques in the cortex, hippocampus, and amygdala. The cingulate gyrus showed severe spongiosis, whereas the medulla oblongata, cervical medulla, and cerebellum showed no abnormalities.

Patient 4 (P4) was admitted to a nursing home at the age of 66 years with a diagnosis of dementia. However, the patient remained alert until her death and continued to recognize people and make demands. An EEG carried out when the patient was 70 years old showed decreased alpha rhythm and an epileptic predisposition although clinically there was no indication of epilepsy. The neuropathology report detailed numerous plaques and tangles across the cortex and hippocampus. The substantia nigra, cingulate gyrus, locus coeruleus, pons, and cerebellum showed no abnormalities. The amygdala showed only a few neurofibrillary tangles and senile plaques.

### 2.2 Tissue processing

Brains were fixed with 0.1 M sodium phosphate–buffered 4% formaldehyde (pH 7.2) for a mean of 8 years for DS brains (range 7–9 years) and 15 years for control brains (range 3–22 years). The cerebrum was detached at the level of the third cranial nerve, the meninges were removed, and hemispheres were processed as previously described (Karlsen and Pakkenberg, 2011). Coronal sections 40-μm thick were stained with Giemsa's azur eosin methylene blue solution (Merck, Darmstadt, Germany) and KHPO_4_ (pH 4.5) in a 1:4 ratio. Slides were coded by a researcher not involved in data collection to allow blinded analyses. Although there were variations in fixation time, there was no obvious difference in tissue quality or counting conditions. Furthermore, length of time in fixative did not correlate with any measure (lowest *p*_c_ = 0.13).

### 2.3 Cell number and volume estimation

**Fig. 1:**
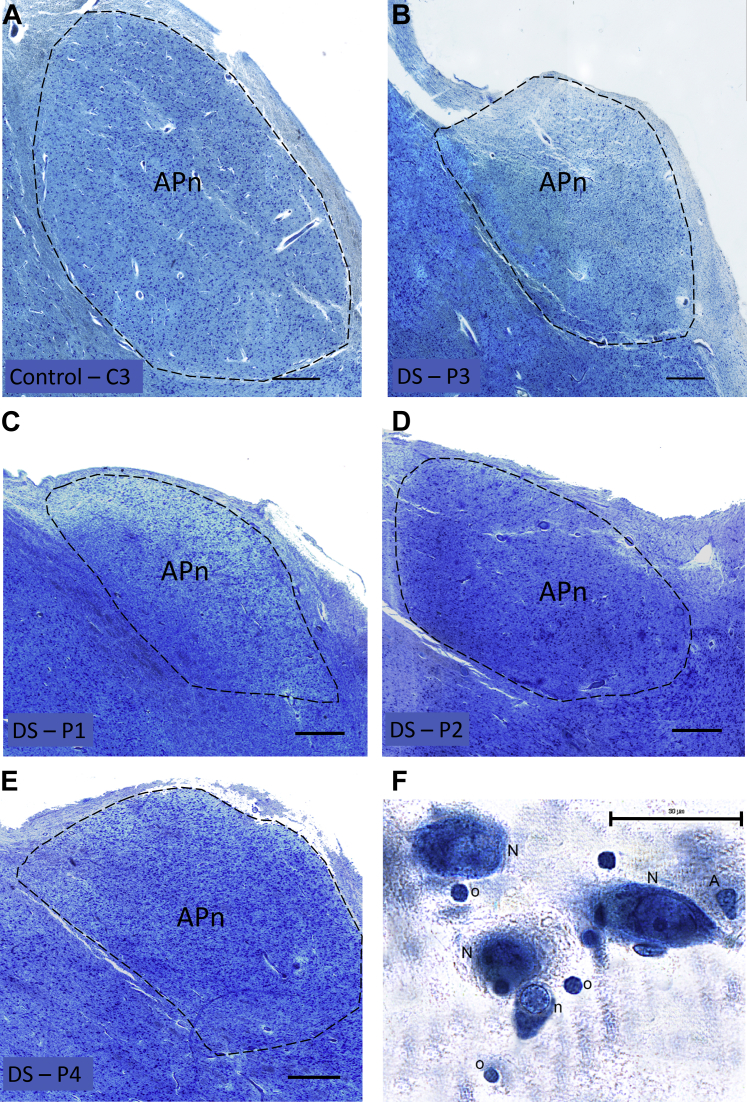
Photomicrographs of the anterior principal thalamic nucleus (APn). Sections were stained with Giemsa's azur eosin methylene blue solution. Image montages of the APn were taken from similar levels from (A) control brain—C3 and (B–E) each of the DS brains. The APn is encapsulated by the internal medullary lamina. Examples of the estimated cell populations (F): N, large (“projection”) neurons; n, small (“inhibitory”) neurons; A, astrocytes; O, oligodendrocytes. Scale bars A–E = 750 μm; Scale bar F = 30 μm. Abbreviation: DS, Down syndrome. (For interpretation of the references to color in this figure legend, the reader is referred to the Web version of this article.)

Owing to the difficulty in distinguishing between the anteromedial and anteroventral thalamic nuclei in Nissl-stained human tissue, these nuclei were grouped together as the anterior principal nuclei (APn; Fig. 1A–E) and delineated as previously described (Armstrong, 1990, Harding et al., 2000, Hirai et al., 1989). The APn is predominantly encapsulated by fibers making it easily identifiable; only the inferior edge of the structure does not have a clear boundary. The APn was distinguishable from the anterodorsal, parataenial, and paraventricular thalamic nuclei by laminae that encapsulate these nuclei. There was a maximum of one section per brain where the central medial thalamic nuclei were not as distinct from the ventral medial tip of the APn. For these sections, where possible, the lamina separating the paraventricular and central medial thalamic nuclei from the APn was used as a guide for distinguishing the ventral medial portion.

The total number of neurons and glial cells were estimated from one hemisphere per brain using the optical fractionator method (Gundersen, 1986, West et al., 1991). Slides were mounted on an Olympus BX60 microscope equipped with a computer-driven X–Y motorized stage (ProScan III Prior, UK) with Z-axis focus control (Heidenhain Encoder, Germany) and a CMOS camera (Basler, Germany) connected to a computer running the NewCast Stereology software package (Visiopharm, Hørsholm, Denmark). Differentiation of neuron and glial cell types was based on cell morphology as previously described (Karlsen and Pakkenberg, 2011, Pelvig et al., 2008). In brief, neurons have a single large nucleolus and a large nucleus surrounded by a visible cytoplasm. Neurons can be further classified into “small” local inhibitory neurons and “large” projecting excitatory neurons based on size (Armstrong, 1990, Dorph-Petersen et al., 2004). As shown in Fig. 1F, large and small neurons are easily distinguishable with small neurons being of comparable size to large astroglia. On the basis of somal size, thalamic neurons show a bimodal frequency distribution with very little overlap between the smallest of the large neurons and the largest of the small neurons (Dewulf, 1971, Dorph-Petersen et al., 2004). These two types of neurons are only referred to as small and large in this study as all cell volumes were biased at least to some degree because of paraffin as embedding media and should be evaluated in that light. Oligodendrocytes were identified as having a small rounded nucleus with dense chromatin and a perinuclear halo (Fig. 1F). Astrocytes have a pale nucleus surrounded by a translucent cytoplasm and heterochromatin concentrated in granules in a rim below the nuclear membrane. Microglia appear small and elongated or comma-shaped with dense peripheral chromatin. The nucleus was the counting item for both neurons and glial cells during sampling.

Sampling of sections followed a systematic, uniform random sampling scheme to ensure all parts of the APn had an equal chance of being sampled. A section sampling fraction of 1/16 was used resulting in 9–15 sections sampled per brain (mounted section thickness 40 μm). The contour of the APn was traced live using a 10× objective (0.4 NA) and cells were counted using a 100× oil immersion objective (NA 1.35) with a final onscreen magnification of 3000×. A disector height of 20 μm with a 5 μm guard zone was used to sample in the z-axis. The counting frame area was 2800 μm^2^ for neurons and 1350 μm^2^ for glial cells. The x–y step length was adjusted to allow optimal sampling of brains considered “large” and “small” but did not change within cases. The x–y step length was set to 400–1000 μm^2^ for neurons and 800–1000 μm^2^ for glial cells. These sampling parameters led to a mean of 206 neurons (range, 152–276) and 631 glial cells (range, 399–1081) counted per brain in a mean of 222 counting frames (range, 131–466) for neurons and 103 counting frames (range, 45–142) for glial cells. The numbers of neurons and glial cells were estimated from

$$N=\frac{1}{ssf}\cdot \frac{1}{asf}\cdot \frac{1}{hsf}\cdot \sum Q^{-}\text{,}$$

where *ssf* is the section sampling fraction; the area sampling fraction (*asf*) is the ratio of the counting frame area to the area of the x–y step length; the height sampling fraction (*hsf*) is the ratio of the disector probe height to the Q^−^ weighted tissue thickness; and ∑ Q^−^ is the total count of cells sampled (Dorph-Petersen et al., 2004). The density of neurons and glia was calculated as N/volume.

The unilateral volume of the APn was estimated according to Cavaleri's principle (Gundersen et al., 1999, Gundersen and Jensen, 1987) but was not corrected for tissue shrinkage as the degree of shrinkage between control and DS brains was estimated to be the same in a previous study using the same samples (Karlsen and Pakkenberg, 2011).

The coefficient of error (CE) was calculated as (Gundersen et al., 1999) to provide information regarding the precision of the estimates. The CE for group estimates of each measure was estimated as

$${\text{CE}}_{x}=\frac{\sqrt{{\text{CE}}_{1}^{2}+{\text{CE}}_{2}^{2}+\ldots +{\text{CE}}_{n}^{2}}}{n}$$

where CE_*x*_ is the mean CE estimate and *n* is the number of individuals. As a measure of the variability of the estimates, the observed interindividual coefficient of variation (CV = standard deviation/mean) are reported in parentheses after group means.

**Fig. 2:**
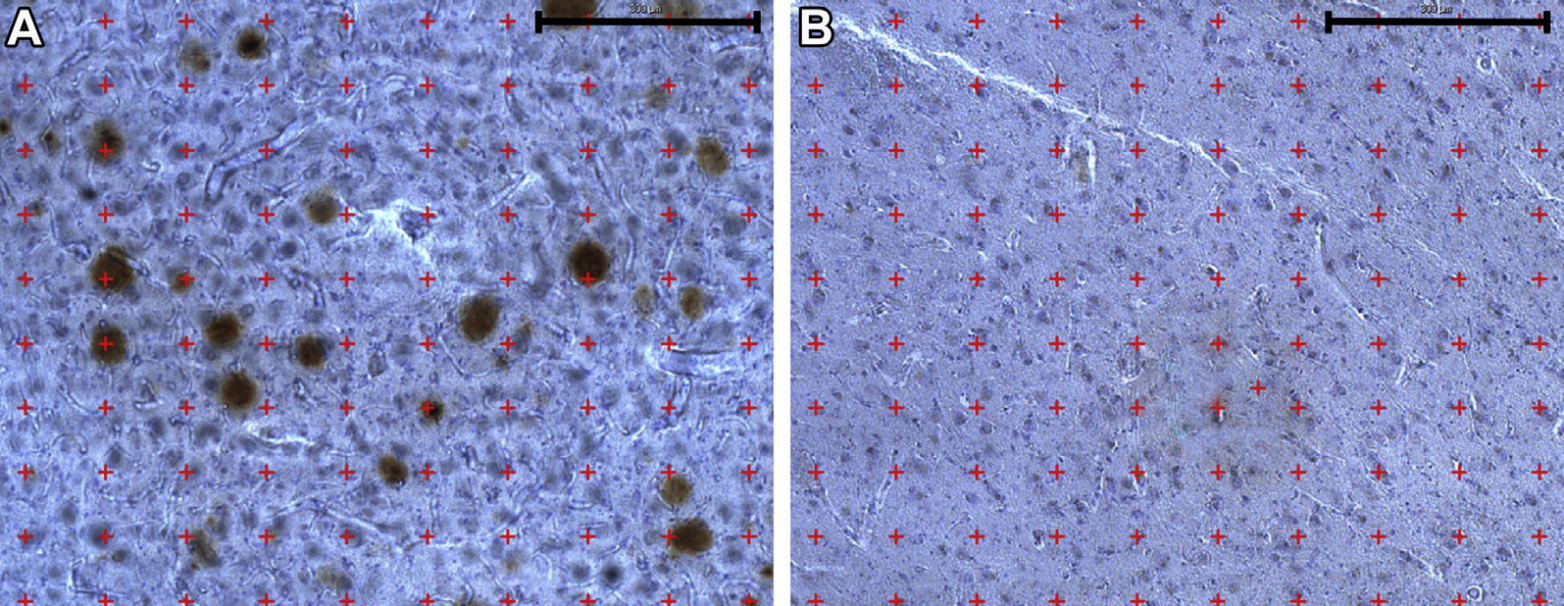
Amyloid-ß (Aß) staining in the anterior principal thalamic nucleus. Example of Aß staining with a grid-overlay (red crosses) for point counting from (A) DS brain–P2 and (B) control brain–C6. Scale bars = 300 μm. Abbreviation: DS, Down syndrome. (For interpretation of the references to color in this figure legend, the reader is referred to the Web version of this article.)

**Fig. 3:**
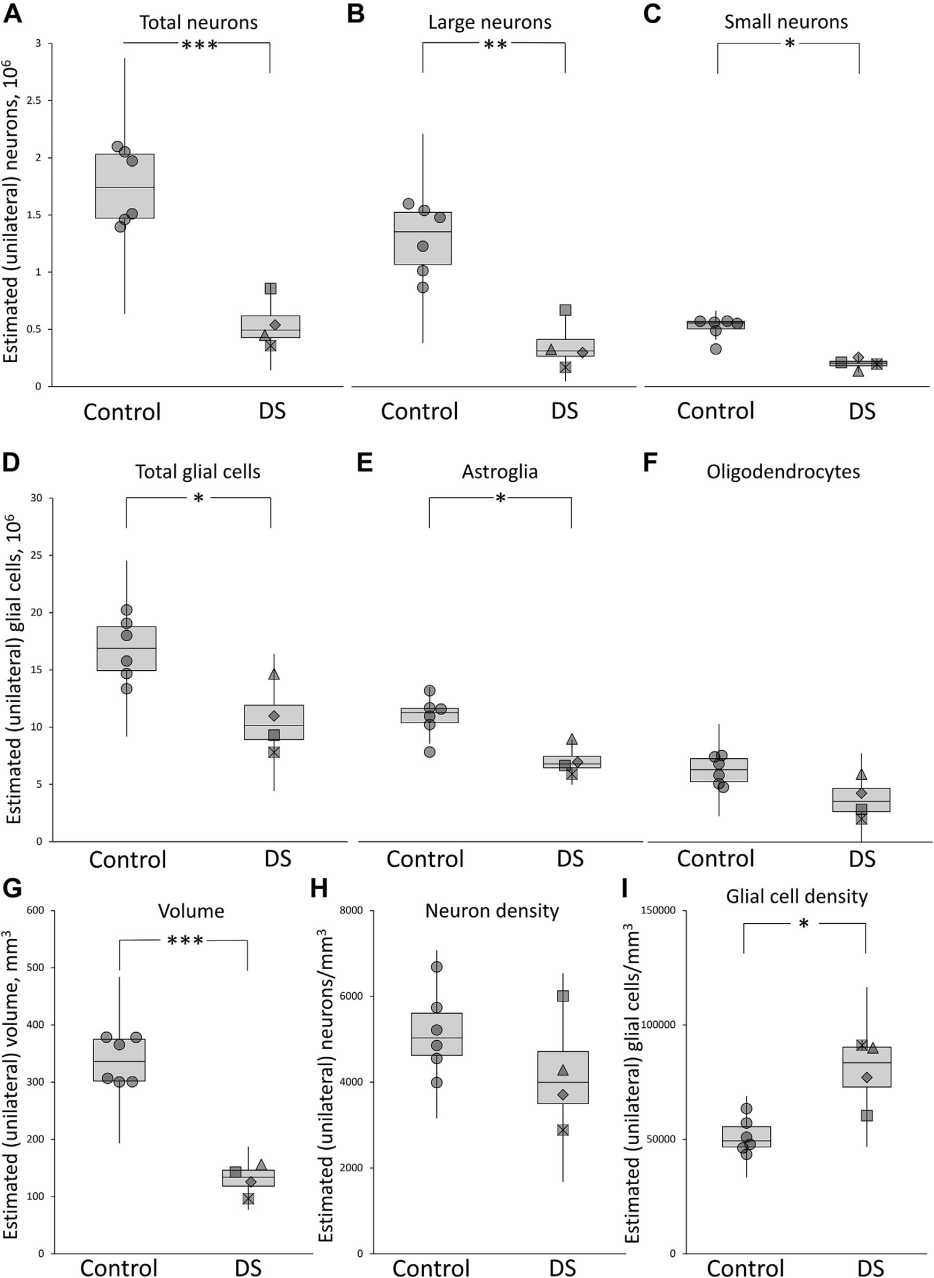
Unilateral estimates for anterior principal thalamic nucleus (APn) in control and Down syndrome (DS) brains. Top panel, (A) total neurons (combined large and small neurons; control = 1.75 × 10^6^ [0.19], DS = 0.55 × 10^6^ [0.39]), (B) large (“projecting”) neurons (control = 1.25 × 10^6^ [0.23], DS = 0.36 × 10^6^ [0.58]), and (C) small (“local inhibitory”) neurons (control = 0.50 × 10^6^ [0.19], DS = 0.20 × 10^6^ [0.24]). Middle panel, (D) total glial cells (combined astroglia, oligodendrocytes, and microglia; control = 16.84 × 10^6^ [0.16], DS = 10.68 × 10^6^ [0.28]), (E) astroglia (control = 10.60 × 10^6^ [0.17], DS = 6.92 × 10^6^ [0.18]), and (F) oligodendrocytes (control = 6.08 × 10^6^ [0.19], DS = 3.67 × 10^6^ [0.45]). Bottom panel, (G) volume of APn (control = 338 mm^3^ [0.12], DS = 130 mm^3^ [0.20]), (H) neuron density (control = 5171 × mm^3^ [0.18], DS = 4221 × mm^3^ [0.31]), and (I) glial cell density (control = 51,509 × mm^3^ [0.14], DS =79,649 × mm^3^ [0.18]). The central line in each box indicates the median value. The box extends from the first to the third quartile range. The whiskers extend 1.5× the interquartile range. Individual data points are shifted along the x-axis to aid visualization of overlapping data points. Note that in F, the whisker for the DS plot extends slightly below zero (not shown). Note that the values in this legend are mean estimates with the coefficient of variation reported in parentheses. Figure symbols refer to DS brain ID# as follows: diamond P1, square with cross P2, triangle P3, and square P4.*Significantly different from control, *p*_c_ < 0.05; **significantly different from control, *p*_c_ < 0.01; ***significantly different from control, *p*_c_ < 0.001.

To provide an indication of the amyloid plaque burden within the APn, 1–2 midsections from DS brains were available for amyloid-ß (Aß) immunohistochemical staining (M0872, 1:1000, DAKO, Denmark). Applying an adapted point-counting method (Madsen et al., 2018), we estimated the area fraction percentage of Aß by overlaying a grid with 100 points spaced equally 115 μm apart (Fig. 2) with a final on-screen magnification of 220×. The number of grid points hitting the Aß plaques and tissue were counted to provide the area fraction of the amyloid plaque burden to tissue.

### 2.4 Statistics

Differences between groups were tested using an independent means *t*-test (two-tailed). Welch's *t*-test for unequal variances was used wherever appropriate. A Mann-Whitney *U* test was used when normality tests failed. Wherever relevant, effect sizes between groups were expressed as Hedges *g* when equal variances were assumed, Glass's Δ when equal variances were not assumed, or η^2^ following a Mann-Whitney *U* test (Fritz et al., 2012). The common language effect size (CL), the percentage of occasions that a randomly sampled case from distribution with the higher mean will have a higher score compared with a randomly sampled case from the distribution with the lower mean (Lakens, 2013, McGraw and Wong, 1992), is also reported for the key measures total neurons, total glial cells, and volume to conceptualize the size of these effects. Pearson's correlation was used to assess relationships between variables. The SPSS software (version 20, IBM Corporation) was used to carry out statistical analyses. The Holm-Bonferroni procedure was used to correct for multiple comparisons. The corrected *p*-values (*p*_c_) of less than 0.05 were considered significant.

The CE estimates for the total number of neurons was 0.07 for both control and DS brains; for large neurons, it was 0.09 for both groups; and for small neurons, it was 0.14 for control and 0.11 for DS brains. The CE estimates for total glial cells was 0.04 for both control and DS brains; for oligodendrocytes, it was 0.07 for control and 0.08 for DS brains; for astroglia, it was 0.05 for both groups; and for microglia, it was 0.51 for controls and 0.49 for DS brains. Estimate of the final CE for volume was 0.05 in both control and DS brains.

## 3 Results

As shown in Fig. 3, there was a 68% reduction in total numbers of neurons in the APn of the DS brains compared with controls. This difference was found to be significant (*t*(7.98) = 6.99, *p*_c_ < 0.001; Δ = 3.68, 95% CI 1.67–5.81, *CL* = 99%; Fig. 3A). This overall reduction reflected changes in both large (*t*(8) = 5.26, *p*_c_ < 0.01; *g* = 3.06, 95% CI 1.22–4.91; Fig. 3B) and small neurons (Mann-Whitney U = 0.00, *p*_c_ = 0.029; η^2^ = 0.65; Fig. 2C).

There was a significant 37% reduction in estimated total number of glial cells in DS brains compared with controls (*t*(8) = 3.43, *p*_c_ = 0.036; *g* = 2.00, 95% CI 1.48–5.43, *CL* = 93%; Fig. 3D). A significant reduction in DS brains was also observed when glial cells were classified as astroglia (t(8) = 3.58, *p*_c_ = 0.036; g = 2.09, 95% CI 0.53–3.65; Fig. 3E). The difference for oligodendrocytes was borderline significant when corrected (*t*(8) = 2.73 *P*_c_ = 0.052, *p* = 0.026; g = 1.59, 95% CI 0.14–3.03; Fig. 3F).

The estimated volume of the APn was 62% less in DS brains than controls and this reduction was significant (*t*(7.99) = 10.09, *p*_c_ < 0.001; Δ = 5.27, 95% CI 2.70–8.05, *CL* = 99%; Fig. 3G). The estimated density of total neurons for DS brains did not differ from the estimate for controls (t(8) = 1.33, *p* = 0.22; Fig. 3H). However, the estimated total glial cell density in DS brains was significantly higher than the estimate in controls, (*t*(8) = 4.12, *p*_c_ = 0.02; *g* = 2.40, 95% CI 0.76–4.05; Fig. 3I). For microglia, the number available for sampling was consistently low (2–20 per brain), resulting in high group CE values (0.49–0.51). Controls were estimated to have 1.64 × 10^6^ (CV, 0.93) microglia, whereas DS brains had 0.94 × 10^6^ (CV, 0.51) microglia, although this reduction was not significant (*p* > 0.40).

The area fraction of the APn occupied by Aß in DS brains had a mean estimate of 7.5% (range, 6%–9%; CV, 0.17). Although no significant correlations between Aß and each measure were found, there was a trend of fewer total neurons (r = −0.62, *P*_c_ > 0.40) and a greater number of total glial cells (r = 0.84, *P*_c_ > 0.40) with increased Aß burden in the APn.

## 4 Discussion

We estimated the total number of neurons and glial cells in the combined anteroventral and anteromedial thalamic nuclei (anterior principal thalamic nuclei, APn) of aged women with DS. When compared with age-matched controls, there were striking changes with almost 70% fewer neurons in the DS brains, with reductions in both large and small neurons. Glial cells were also reduced but to a lesser degree (37%), with comparable changes across astrocytes and oligodendrocytes. The overall volume of APn was also markedly reduced (62%) in patients with DS. As a result, there was no difference in neuronal density between patients with DS and controls. A similar pattern is found in Korsakoff patients whereby neuron density is unaffected but overall numbers are reduced by ∼50% (Harding et al., 2000), highlighting the importance of assessing overall cell counts and not just neuronal density.

While the present study focused on the APn, previous studies using the same brains have reported changes in the mediodorsal thalamic nucleus and cortical regions (Karlsen et al., 2014, Karlsen and Pakkenberg, 2011). While the volume of the mediodorsal thalamic nucleus was reduced to a similar extent as the APn (59%), the overall cell loss was less with a 43% reduction in neurons but no changes in glial cells (Karlsen et al., 2014). This highlights the limitations of treating the thalamus as a unitary structure. Furthermore, it becomes clear that simple volumetric measures can mask substantial variations in patterns of cell loss. Compared with the cortex, there were greater reductions in the APn when considering both volume and neuronal count reduction (i.e., ∼40% for both in cortex; Karlsen and Pakkenberg, 2011). By contrast, the reduction in neocortical glial cells was more comparable with the current findings (i.e., ∼30%; Karlsen and Pakkenberg, 2011). Together, the changes found in APn are more pronounced than other areas assessed in the same brains, particularly when considering the reduction in neurons.

While there are some differences between the patient and control groups in terms of the cause of death, postmortem interval, and length of time in fixative (see Table 1), the present findings are unlikely to be because of these factors (Blair et al., 2016). The basal ganglia were also examined in these same brains and there was no neuronal loss in this structure (Karlsen and Pakkenberg, 2011), confirming the specificity of the current findings.

An obvious consideration is the extent to which these APn changes simply reflect the accompanying dementia in this patient group rather than being directly related to DS, especially given the finding that neuronal numbers within some thalamic nuclei have been shown to correlate with amyloid levels (Erskine et al., 2016, Erskine et al., 2017). Pathology related to AD is a common feature in patients with DS with the onset of amyloid pathology commencing as early as the late teens and dementia often occurring by the mid 50s (Davidson et al., 2018, Head et al., 2012). Across the cortex and hippocampal formation, the development patterns of neurofibrillary tangles are comparable across DS and AD groups although there appears to be more widespread and denser deposition of amyloid plaques in DS (Hof et al., 1995). However, Braak found no noticeable difference in AD-related pathology in the anterior thalamus of patients with AD with or without DS (Braak and Braak, 1991a). There are surprisingly few studies that have quantitatively assessed the ATN in AD (without accompanying DS) and even fewer that have carried out neuronal counts. Although tangles and plaques are present in the APn, it is the anterodorsal thalamic nucleus that is most severely affected (Braak and Braak, 1991a, Rub et al., 2016, Xuereb et al., 1991). Consistent with this, Xuereb et al. (1991) found no significant reduction in either volume or neuronal counts in the APn of patients with AD, although there was a significant reduction in neuronal counts in the anterodorsal nucleus. Similarly, Hornberger et al. (2012) found no significant reduction in ATN volume in patients with AD using either neuroimaging or postmortem tissue. Given the small cohort, it is difficult to draw conclusions regarding AD pathology in the present study. Two of the patients were categorized as Braak stage 5 and two as Braak stage 6, which relates to the extent of neurofibrillary pathology in the brain. These are both considered late stages of AD with abnormal tau pathology extending into the nonlimbic cortical areas (Braak et al., 2006). There is no clear relationship between Braak stage of neurofibrillary tangles and APn volume or neuronal density from the current data. By contrast, the number of glia cells in the stage 6 patients seems higher than those in stage 5 patients, especially when considering oligodendrocytes. All patients appeared to have the most severe Braak staging of amyloid (i.e., “c”), where amyloid deposits are found across cortical areas (Braak and Braak, 1991b). Accordingly, our semiquantitative analysis of amyloid protein found high levels in the APn in the current patients with a (nonsignificant) trend of fewer neurons and a greater number of glial cells with a greater amyloid presence. Taken together, the APn pathology we report here does not seem to mirror what is found in standard AD, but the earlier onset of AD-like pathology in DS and the greater presence of amyloid plaques may exacerbate the cell loss. Surprisingly, the current findings show greater similarity to other dementias such as the semantic variant of primary progressive aphasia and frontotemporal dementia where there is ∼30%–40% decrease in ATN volume (Tan et al., 2014).

The current findings raise the question as to whether ATN pathology is a common feature of DS and whether the ATN are also compromised in younger patients with DS. At present, very little is known about the status of the ATN in this patient group. Studies in younger patients have typically only measured the thalamus as a whole and in doing so have not found any difference between patient and control groups (Annus et al., 2017; but see; Gunbey et al., 2017, Jernigan et al., 1993, Weis et al., 1991). However, this approach may be masking more selective changes within the ATN. Reports of enlarged 3rd ventricles in younger patients with DS could also be indicative of atrophy within the ATN (Raz et al., 1995, Schimmel et al., 2006). Consistent with this, the mammillary bodies were shown to be markedly smaller in adults with DS; moreover, their size significantly correlated with general aptitude (Raz et al., 1995). Given the reductions in mammillary body and hippocampal volume, and the dense connectivity between these structures and the ATN (Bubb et al., 2017), it is possible that the ATN are also compromised at an earlier time point in patients with DS. Pathology within the extended hippocampal-diencephalic network would be consistent with the pattern of memory impairments observed in DS, where there is greater disruption to explicit compared with implicit memory (Vicari et al., 2000). A similar memory profile is found after damage to the mammillary body-thalamic axis (Carlesimo et al., 2007, Tsivilis et al., 2008, Vann et al., 2009).

A limitation of the study is the small sample size, as is often the case with postmortem studies. However, the pattern of cell loss in all DS brains was very consistent, both in the APn as well as previous structures measured (Karlsen et al., 2014, Karlsen and Pakkenberg, 2011). Furthermore, although we had a low sample size, the differences found on key measures was reliable, with large effect sizes. The sample size was, therefore, sufficient to detect the differences reported, although there are clear limitations in the correlative inferences that can be made. Small sample sizes can also produce erroneous effects because of incomplete sampling of the population; again the consistency in findings across various measures makes this less likely to be the case in the current study. There was also limited information available regarding premortem cognitive status although from the information available the least cognitively impaired patient (P4) had the greatest number of APn neurons.

Together, these data show a striking decrease in both the volume and cell number of APn in aged patients with DS. The pathology in this region is likely to contribute to memory impairments given the importance of this region for mnemonic processes. These findings reinforce the need to look beyond the medial temporal lobe when considering neurological conditions that present with memory impairments. There is also a clear need to further assess this structure in future studies of DS to determine the age of onset of ATN pathology in this patient group.

## Disclosure

The authors have no actual or potential conflicts of interest.
